# Suicidal Ideation during the COVID-19 Pandemic among A Large-Scale Iranian Sample: The Roles of Generalized Trust, Insomnia, and Fear of COVID-19

**DOI:** 10.3390/healthcare10010093

**Published:** 2022-01-04

**Authors:** Chung-Ying Lin, Zainab Alimoradi, Narges Ehsani, Maurice M. Ohayon, Shun-Hua Chen, Mark D. Griffiths, Amir H. Pakpour

**Affiliations:** 1 Institute of Allied Health Sciences, National Cheng Kung University Hospital, College of Medicine, National Cheng Kung University, Tainan 701, Taiwan; cylin36933@gmail.com; 2 Social Determinants of Health Research Center, Research Institute for Prevention of Non-Communicable Diseases, Qazvin University of Medical Sciences, Qazvin 3419759811, Iran; zainabalimoradi@yahoo.com (Z.A.); narges.ehsany@yahoo.com (N.E.); 3 Stanford Sleep Epidemiology Research Center (SSERC), School of Medicine, Stanford University, Stanford, CA 94305, USA; mohayon@stanford.edu; 4 School of Nursing, Fooyin University, Kaohsiung 83102, Taiwan; 5 International Gaming Research Unit, Psychology Department, Nottingham Trent University, Nottingham NG1 4FQ, UK; mark.griffiths@ntu.ac.uk; 6 Department of Nursing, School of Health and Welfare, Jönköping University, 55111 Jönköping, Sweden

**Keywords:** generalized trust, psychological distress, sleep, insomnia, suicide

## Abstract

The novel 2019 coronavirus disease (COVID-19) is still not under control globally. The pandemic has caused mental health issues among many different cohorts and suicidal ideation in relation to COVID-19 has been reported in a number of recent studies. Therefore, the present study proposed a model to explain the associations between generalized trust, fear of COVID-19, insomnia, and suicidal ideation during the COVID-19 pandemic among a large-scale Iranian sample. Utilizing cluster sampling with multistage stratification, residents from Qazvin province in Iran were invited to participate in the present study. Adults aged over 18 years (n = 10,843; 6751 [62.3%] females) completed ‘paper–and-pencil’ questionnaires with the assistance of a trained research assistant. Structural equation modeling (SEM) was applied to understand the associations between generalized trust, fear of COVID-19, insomnia, and suicidal ideation. Slightly over one-fifth of the participants (n = 2252; 20.8%) reported suicidal ideation. Moreover, the SEM results indicated that generalized trust was indirectly associated with suicidal ideation via fear of COVID-19 and insomnia. Furthermore, generalized trust was not directly associated with suicidal ideation. The proposed model was invariant across gender groups, age groups, and participants residing in different areas (i.e., urban vs. rural). Generalized trust might reduce individuals’ suicidal ideation during the COVID-19 pandemic period via reduced levels of fear of COVID-19 and insomnia. Healthcare providers and policymakers may want to assist individuals in developing their generalized trust, reducing fear of COVID-19, and improving insomnia problems to avoid possible suicidal behaviors.

## 1. Introduction

The psychological distress induced by the novel coronavirus disease-2019 (COVID-19) has had a large negative impact across different countries and regions worldwide [1,2,3,4,5,6]. With such long-lasting suffering, it is likely that some individuals develop various severe mental health problems, including suicidal ideation [7]. A recent paper reported that in Bangladesh during the pandemic, the prevalence of suicidal ideation was 5% during 1 to 10 April 2020, 6% during 8 to 25 April 2020, 12.8% during April to May 2020, and 19.0% during July 2020 [8].

Moreover, constructing the relationships between these factors is needed to help build a theoretical model to guide potential treatment directions for target outcomes [9,10]. In the present study, a proposed model was constructed to provide knowledge concerning the underlying psychological mechanisms for healthcare providers to understand how suicidal ideation is generated. In this regard, three important factors (i.e., generalized trust, fear of COVID-19, and insomnia) are proposed to be important factors contributing to suicidal ideation during the COVID-19 pandemic [11,12,13,14,15,16].

Generalized trust (which is also called ‘general trust’ or simply ‘trust’ by some researchers) is defined as “a willingness to be vulnerable to the actions of others” [17] and is found to be an important societal factor in assisting individuals in maintaining daily living [18]. Generalized trust has been found to be a factor that contributes to good social relationships, which may subsequently improve individuals’ health via the mutual trust and positive interactions between individuals [19,20,21]. Consequently, individuals’ psychological health can be improved [22,23,24,25]. Therefore, it is hypothesized that generalized trust during the COVID-19 pandemic may help reduce individuals’ fear of COVID-19, insomnia, and suicidal ideation.

A systematic review shows that the suicidal ideation and behaviors during the COVID-19 pandemic were increased compared with the period before the COVID-19 pandemic [8]. Moreover, evidence has shown that some suicide attempts are related to fear of COVID-19 or worries about COVID-19 [11,16]. For example, the first suicide case in Bangladesh involved a man who killed himself because he experienced COVID-19-like symptoms, although the autopsy showed he was not infected with COVID-19 [11]. In another study, fear of COVID-19 was significantly associated with suicidal ideation among 1013 English-speaking Americans [16]. Similarly, it was found that fear of COVID-19, together with psychological distress, explained 27% of the risk regarding suicidal ideation among 595 Spanish adults [26]. The relationship between fear and suicidal ideation has also been found in another coronavirus (i.e., SARS) [27]. Therefore, in extreme cases, fear of COVID-19 may trigger an individual’s irrational thoughts and result in suicide.

Aside from fear of COVID-19, insomnia has been found to be another important factor in contributing to individuals’ suicidal ideation. Before the COVID-19 pandemic, there was evidence that insomnia severity was a useful indicator in predicting suicidal ideation [13]. Moreover, prior research, including randomized controlled trials, has shown that tackling insomnia can substantially reduce individuals’ suicidal ideation [12,14]. There is also some evidence that insomnia has been associated with suicidal ideation during the pandemic [16]. Moreover, sleep problems during the COVID-19 pandemic have been found to be serious [28,29]. Therefore, insomnia could be a trigger for individuals to develop suicidal ideation during the COVID-19 pandemic.

To the best of the present authors’ knowledge, the contemporary evidence has supported the associations between fear of COVID-19, insomnia, and suicidal ideation during the COVID-19 pandemic [16,26]. However, there is a lack of empirical evidence regarding how generalized trust is associated with fear of COVID-19, insomnia, and suicidal ideation during the COVID-19 pandemic period. However, prior to the COVID-19 pandemic, generalized trust was found to be associated with low levels of fear, insomnia, and suicidal ideation. Therefore, the present study proposed a model that adopts generalized trust as the independent variable, fear of COVID-19 and insomnia as mediating variables, and suicidal ideation as the dependent variable. The proposed model was examined among a large-scale Iranian sample to provide evidence regarding the psychological mechanism underlying generalized trust and suicidal ideation. More specifically, the present study investigated whether insomnia and fear of COVID-19 are mediators in the association between generalized trust and suicidal ideation among the Iranian general population during the COVID-19 pandemic.

## 2. Materials and Methods

### 2.1. Procedures and Participants

The target population in the present study was the general population from Qazvin province in Iran, including residents who lived in urban or rural areas during the COVID-19 pandemic. The entire population residing in Qazvin, a province located in the central part of Iran, comprised 1,273,761 individuals in 2018 with 51% of the Qazvin population being male. The present study used cluster sampling with multistage stratification to recruit participants. First, 70 strata were stratified according to the administration districts in Qazvin. Second, several health centers in each stratum were randomly selected with the consideration of the population size in the stratum. Third, a list of families in the health centers was provided by each health center and several families were randomly selected. Fourth, interviewers who had received formal training contacted the selected families and introduced the study to them. More specifically, the participants’ rights and autonomy were clearly described. Once eligible individuals were willing to participate, an interviewer performed a home visit to describe further study details to the participant. If they provided written informed consent, they were allowed to complete the survey.

There was no obvious pattern of selection bias and there was a relatively high response rate (78%). The present study recruited a sample that was considered to be representative of the general adult population in Qazvin. The survey period was administered between 19 February and 9 April 2021. In order to maximize the representativeness of the general adult population, there were only two inclusion criteria. These were being (i) an adult aged 18 years or older living in Qazvin, and (ii) an adult who provided written informed consent for participation. Guests and tourists in Qazvin were excluded from the study.

### 2.2. Measures

Suicidal ideation was assessed using Item 9 from the nine-item Patient Health Questionnaire (PHQ-9). The PHQ-9 suicide item has been found to be an effective tool for assessing the prevalence of suicidal ideation. The PHQ-9 suicide item assesses passive thoughts of death and the desire for self-harm among respondents (i.e., “Thoughts that you would be better off dead, or thoughts of hurting yourself in some way?”) within the last two weeks. The Iranian version of the PHQ-9 has been found to be valid and reliable [30].

General trust was assessed using the six-item Generalized Trust Scale (GTS) [21,31]. All six items (e.g., “Most people are basically honest”) are rated on a five-point scale with a higher score indicating a higher level of generalized trust [21,31]. The GTS was recently translated into Persian for Iranians and demonstrated satisfactory psychometric properties (Cronbach’s α = 0.89 and is unidimensional among the Iranian general population [18,32]).

Fear of COVID-19 was assessed using the seven-item Fear of COVID-19 Scale (FCV-19S) [33]. All seven items (e.g., “I am most afraid of coronavirus-19”) are rated on a five-point scale with a higher score indicating a higher level of COVID-19 fear [34,35]. The FCV-19S was originally developed in Persian among the Iranian general population and the scale demonstrated satisfactory psychometric properties (Cronbach’s α = 0.82 and is unidimensional among the Iranian general population [33]).

Insomnia was assessed using the seven-item Insomnia Severity Index (ISI) [36]. All seven items (e.g., “How satisfied/dissatisfied are you with your current sleep pattern?”) are rated on a five-point scale with a higher score indicating a higher level of insomnia [37,38]. The ISI has been translated into Persian for Iranians and the Persian ISI demonstrated satisfactory psychometric properties (Cronbach’s α between 0.82 and 0.87 among Iranian patients with insomnia [39] and is unidimensional among Iranian patients with cancer [37]).

Demographic information was collected using a background information sheet including their age (in years), sex (male or female), educational status (university, diploma, high school, secondary school, primary school, or no formal education), marital status (married or single), and accommodation (city or rural).

### 2.3. Data Analysis

Descriptive statistics were first carried out to understand the participants’ characteristics, including their age, sex, educational status, marital status, accommodation, insomnia, fear of COVID-19, and generalized trust. Moreover, the frequency of suicidal ideation was calculated for the sample and different subgroups of the sample (e.g., male and female). Then, Pearson correlations were conducted to examine the zero-order correlations between the studied variables in the proposed model (i.e., suicidal ideation, insomnia, fear of COVID-19, and generalized trust). The proposed model in the present study was then evaluated using structural equation modeling (SEM) with full information maximum likelihood.

In the SEM, all the instrument scores were summed to be used as an observed variable in the proposed model to fulfill the principle of parsimony. The proposed model was first evaluated using fit indices of nonsignificant χ^2^, comparative fit index (CFI), and Tucker–Lewis index (TLI) > 0.9, together with root mean square error of approximation (RMSEA) and standardized root mean square residual (SRMR) < 0.08 [40,41,42]. When the proposed model shows satisfactory fit, the mediated effects of insomnia and fear of COVID-19 in the association between generalized trust and suicidal ideation were checked using the bootstrapping method. More specifically, 5000 bootstrapping samples were generated. Then, the lower and upper limits in the 95% confidence interval (CI) were calculated and the bootstrapping samples were adopted to evaluate the mediated effects. When both limits of the 95% CI do not include 0 (i.e., both are negative values or both are positive values), the mediated effect is supported [43].

The proposed model was then evaluated for its invariance across different subgroups, including gender group (males vs. females), age group (mean age above 35.54 years vs. mean age below 35.54 years), and accommodation group (urban residents vs. rural residents). Multigroup SEM was used to examine the invariance with four nested models; M1: configural model which did not constrain any parameters in the proposed model; M2: a model based on M1 to constrain path coefficients in the proposed model being equal across subgroups; M3: a model based on M2 to constrain correlation coefficients in the proposed model being equal across subgroups; and M4: a model based on M3 to constrain residuals in the proposed model being equal across subgroups. Invariance was supported when ∆CFI (i.e., the difference of CFI between every two nested models) > −0.01, ∆SRMR (i.e., the difference of SRMR between every two nested models) < 0.03, and ∆RMSEA (i.e., the difference of RMSEA between every two nested models) < 0.015 [44].

### 2.4. Patient and Public Involvement Statement

Study participants and the public were not involved in the design, conduct, reporting, or dissemination plans of the present study.

### 2.5. Ethical Consideration

The Ethics Committee of Qazvin University Medical Sciences approved the study procedures (reference number IR.QUMS.REC.1399.418).

## 3. Results

In the large-scale sample of 10,843 participants (mean age = 35.54 years; SD = 12.00), 6751 were females (62.3%), and 2252 reported suicidal ideation (20.8%). The present sample was well educated with over half of them (n = 6991) having a diploma or higher degree (64.5%). Nearly three-quarters of the participants were married (n = 8092; 74.6%) and slightly more than three-quarters resided in a city (n = 8187; 75.5%). A higher prevalence of suicidal ideation was found among those who (i) were female, (ii) had a lower educational level, (iii) were married, and (iv) were living in a city (Table 1). The participants’ levels of insomnia, fear of COVID-19, and generalized trust performances are also presented in Table 1.

Suicidal ideation was positively and significantly correlated with insomnia (r = 0.327; *p* < 0.001) and fear of COVID-19 (r = 0.353; *p* < 0.001), and negatively and significantly correlated with generalized trust (r = −0.070; *p* < 0.001). Generalized trust was negatively and significantly correlated with insomnia (r = −0.100; *p* < 0.001) and fear of COVID-19 (r = −0.183; *p* < 0.001). Moreover, insomnia and fear of COVID-19 were positively and significantly correlated (r = 0.271; *p* < 0.001).

**Figure 1:**
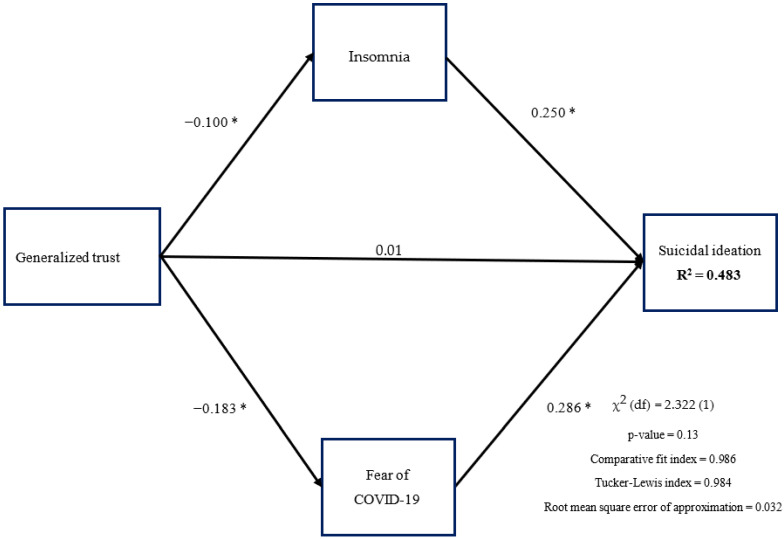
Hypothesized mediation model and findings. * *p* < 0.05.

The proposed model was supported by the fit indices, including CFI (0.986), TLI (0.984), RMSEA (0.032), SRMR (0.028), and the nonsignificant χ^2^ test (*p* = 0.13). In addition, generalized trust had direct effects on insomnia (standardized coefficient [β] = −0.100; *p* < 0.001) and fear of COVID-19 (β = −0.183; *p* < 0.001) but not on suicidal ideation (β = 0.007; *p* = 0.393). Insomnia (β = 0.250; *p* < 0.001) and fear of COVID-19 (β = 0.286; *p* < 0.001) both had direct effects on suicidal ideation (Figure 1). The mediated effects of insomnia and fear of COVID-19 in the association between generalized trust and suicidal ideation were supported by the 95% CI of bootstrapping samples (Table 2).

The proposed model was then tested for its invariance across different subgroups. The multigroup SEM showed that the path coefficients in the proposed model were invariant across gender groups (∆CFI = 0.002 to 0.006; ∆RMSEA = −0.004 to −0.001; ∆SRMR = −0.002 to 0.000), age groups (∆CFI = 0.001 to 0.004; ∆RMSEA = −0.006 to −0.003; ∆SRMR = −0.003 to −0.001), and type of residence (∆CFI = 0.001 to 0.005; ∆RMSEA = −0.002; ∆SRMR = −0.005 to 0.000) in terms of path coefficients, correlations, and residuals (Table 3).

## 4. Discussion

The present study used a large sample of the Iranian general public to examine the associations between generalized trust, fear of COVID-19, insomnia severity, and suicidal ideation. The findings indicated that the proposed mediation model (i.e., fear of COVID-19 and insomnia as mediators in the association between generalized trust and suicidal ideation) was supported by the SEM fit indices. Moreover, both mediated effects derived from fear of COVID-19 and insomnia were significant, whereas the direct effect between generalized trust and suicidal ideation was non-significant. The proposed mediation model was further found to be invariant across the three examined subgroups: males vs. females, younger participants vs. older participants, and participants residing in an urban area vs. participants residing in a rural area.

Generalized trust was found to be associated with suicidal ideation indirectly via fear of COVID-19 and insomnia but not directly associated with suicidal ideation. Yamamura (2015) used a survey dataset that was representative of the Japanese general public (i.e., Japanese General Social Survey) and found that high generalized trust can deter suicidal ideation [15]. However, to the best of the present authors’ knowledge, it is unclear why generalized trust is associated with reduced suicidal ideation. Therefore, the present study’s findings provide the possible mechanism in the association between generalized trust and suicidal ideation during the COVID-19 pandemic. That is, generalized trust may decrease individuals’ fear of COVID-19 and insomnia, both of which are important predictors of suicidal ideation [12,13,14,16]. Moreover, existing evidence indicates that generalized trust can help individuals decrease psychological distress and improve psychological health [22,23,24,25]. Therefore, higher levels of generalized trust may reduce individuals’ fear of COVID-19 and insomnia during the COVID-19 pandemic, resulting in lower suicidal ideation among individuals.

Fear of COVID-19 and insomnia are potential factors contributing to individuals’ suicide attempts. Completed suicides during the COVID-19 pandemic were reported and one of the most salient reasons for these individuals committing suicide was the fear of COVID-19 [11,45]. Moreover, individuals who have been exposed to environments with a high risk of COVID-19 infection are likely to have stronger suicidal ideation. This can be explained by worrying that they might transmit their own COVID-19 infection to their loved ones [45]. Regarding the association between insomnia and suicidal ideation, strong evidence from randomized controlled trials has supported the causal effects between insomnia and suicidal ideation (i.e., when individuals’ sleep quality improves, their suicidal thoughts are reduced [12,13,14]). Therefore, the findings of the present study echo prior literature regarding the relationship between insomnia and suicidal ideation.

Based on the present study’s results, there are several clinical implications. Policymakers should implement policies that can improve generalized trust among society members, especially utilizing educational programs. However, given that the increase of generalized trust takes time, other strategies are needed to tackle the emergent needs during the COVID-19 pandemic. Therefore, healthcare providers need to design fear reduction programs or insomnia improvement programs (e.g., cognitive behavioral therapy for insomnia) to reduce the levels of fear of COVID-19 and insomnia severity among individuals [12,13,14]. Moreover, such programs should be converted into online versions to increase the feasibility of success [46]. Programs on reducing fear may work on pervasive anxiety given that this type of anxiety seems to be an important factor contributing to low generalized trust. In other words, when pervasive anxiety with COVID-19 is reduced in general, it is possible to increase generalized trust in the general population. Although it is postulated that pervasive anxiety is an important factor that may contribute to low generalized trust, the present study does not have any evidence showing the associations between pervasive anxiety and generalized trust. Therefore, future studies are encouraged to further probe this issue.

There are some limitations in the present study. First, the present study used a cross-sectional design to examine the associations between generalized trust, fear of COVID-19, insomnia, and suicidal ideation. Although the relationships between these factors are supported by potential mechanisms and previous empirical research, the present findings only provide empirical evidence concerning associations rather than causality between these factors. Therefore, future studies are warranted to provide stronger evidence (e.g., using a longitudinal study design) to verify the model proposed in the present study. Second, the present sample was recruited from Iran, a country that substantially values harmony and family. Therefore, it is unknown whether the present findings can be applied to a country that values individualism. Third, the severity of the COVID-19 outbreak was different across countries worldwide [47,48]. Therefore, the present findings may not generalize to other countries with different severity levels of COVID-19 than Iran.

## 5. Conclusions

The present study suggests that generalized trust might reduce levels of fear of COVID-19 and insomnia; then, the reduced levels of fear of COVID-19 and insomnia might decrease suicidal ideation. Therefore, healthcare providers may want to find ways to improve generalized trust to resolve the mental health problems during the COVID-19 pandemic. For example, helping people cope with pervasive anxiety, a potentially important factor influencing individuals’ generalized trust, may be a solution.

## Figures and Tables

**Table 1:** The demographic characteristics of study participants (n = 10,843).

	Mean ± SD or N (%)	Suicidal Ideation	
		No (n = 8591)	Yes (n = 2252)
Age	35.54 ± 12.00		
Sex			
Female	6751 (62.3%)	5242 (77.6%)	1509 (22.4%)
Male	4092 (37.7%)	3349 (81.8%)	743 (18.2%)
Educational status			
University	4230 (39.0%)	3423 (80.9%)	807 (19.1%)
Diploma	2761 (25.5%)	2180 (79.0%)	581 (21.0%)
High school	974 (9.0%)	768 (78.9%)	206 (21.1%)
Secondary school	1540 (14.2%)	1192 (77.4%)	348 (22.6%)
Primary school	986 (9.1%)	760 (77.1%)	226 (22.9%)
No formal education	352 (3.2%)	268 (76.1%)	84 (23.9%)
Marital status			
Married	8092 (74.6%)	6343 (78.4%)	1749 (21.6%)
Single	2751 (25.4%)	2248 (81.7%)	503 (18.3%)
Accommodation			
City	8187 (75.5%)	6476 (79.1%)	1711 (20.9%)
Rural	2656 (24.5%)	2115 (79.6%)	541 (20.4%)
Insomnia	8.69 ± 5.47		
Fear of COVID-19	21.10 ± 6.95		
Generalized trust	2.81 ± 0.87		

**Table 2:** Models that tested mediated effects of fear and insomnia.

	Stand. Coeff.	Unstand. Coeff.	Bootstrapping SE	Bootstrapping LLCI	Bootstrapping ULCI	*p*-Value
Total effect of generalized trust on suicidal ideation	−0.07	−0.032	0.004	−0.041	−0.024	<0.001
Direct effect of generalized trust on suicidal ideation	0.007	0.003	0.004	−0.004	0.011	0.393
Direct effect of generalized trust on mediators						
Fear of COVID-19	−0.183	−1.453	0.082	−1.618	−1.297	<0.001
Insomnia	−0.100	−0.860	0.085	−1.030	−0.694	<0.001
Indirect effect of generalized trust on suicidal ideation						
Total indirect effect	−0.078	−0.036	0.002	−0.041	−0.032	<0.001
Through fear of COVID-19	−0.045	−0.021	0.001	−0.024	−0.018	<0.001
Through insomnia	−0.013	−0.006	0.001	−0.009	−0.004	<0.001

Note: age, gender, education, accommodation, and marital status were adjusted for the model. Unstand. Coeff. = unstandardized coefficient; LLCI = lower limit in 95% confidence interval; ULCI = upper limit in 95% confidence interval.

**Table 3:** Path invariance across age, gender, and living place through multigroup structural equation modeling analysis.

Model and Comparisons	Fit Statistics									
	χ^2^ (df)/*p*	∆χ^2^ (∆df)/*p*	CFI	∆CFI	TLI	∆TLI	RMSEA	∆RMSEA	SRMR	∆SRMR
Gender (males vs. females)										
M1: Unconstrained	7.40 (2)/0.02	-	0.981	-	0.979	-	0.032	-	0.038	-
M2: Structural weights	13.65 (7)/0.06	6.25 (5)/0.28	0.987	0.006	0.980	0.001	0.028	−0.004	0.037	−0.001
M3: Structural covariances	15.14 (8)/0.06	7.74 (6)/0.26	0.989	0.002	0.982	0.002	0.027	−0.001	0.037	0.000
M4: Structural residuals	18.59 (11)/0.07	11.19 (9)/0.26	0.991	0.002	0.985	0.003	0.024	−0.003	0.035	−0.002
Age (>35.54 years vs. <35.54 years)										
M1: Unconstrained	7.68 (2)/0.02	-	0.980	-	0.978	-	0.033	-	0.039	-
M2: Structural weights	12.81 (7)/0.08	5.13 (5)/0.40	0.984	0.004	0.983	0.005	0.027	−0.006	0.037	−0.002
M3: Structural covariances	13.60 (8)/0.09	5.92 (6)/0.43	0.985	0.001	0.984	0.001	0.022	−0.005	0.036	−0.001
M4: Structural residuals	17.32 (11)/0.10	9.64 (9)/0.38	0.988	0.003	0.986	0.002	0.019	−0.003	0.033	−0.003
Living (city vs. rural)										
M1: Unconstrained	7.01 (2)/0.03	-	0.982	-	0.980	-	0.030	-	0.036	-
M2: Structural weights	13.81 (7)/0.055	6.80 (5)/0.24	0.983	0.001	0.982	0.002	0.028	−0.002	0.036	0.000
M3: Structural covariances	14.18 (8)/0.08	7.17 (6)/0.31	0.984	0.001	0.983	0.001	0.026	−0.002	0.035	−0.001
M4: Structural residuals	16.80 (11)/0.11	9.79 (9)/0.37	0.989	0.005	0.987	0.004	0.024	−0.002	0.030	−0.005

CFI = comparative fit index; TLI = Tucker–Lewis index; RMSEA = root mean square error of approximation; SRMR = standardized root mean square residual.

## Data Availability

The datasets used for this research on adolescents cannot be shared with the public as per the privacy policy and regulations of Qazvin University of Medical Sciences.
